# Unlearning Incorrect Associations in Word Learning: Evidence From Eye‐Tracking

**DOI:** 10.1111/cogs.70077

**Published:** 2025-06-11

**Authors:** Tanja C. Roembke, Bob McMurray

**Affiliations:** ^1^ Institute of Psychology RWTH Aachen University; ^2^ Department of Psychological and Brain Sciences, Department of Communication Sciences and Disorders, Department of Linguistics University of Iowa

**Keywords:** Word learning, Pruning, Vocabulary acquisition, Negative associative learning

## Abstract

Computational and animal models suggest that the unlearning or pruning of incorrect meanings matters for word learning. However, it is currently unclear how such pruning occurs during word learning and to what extent it depends on supervised and unsupervised learning. In two experiments (*N*_1_ = 40; *N*_2_ = 42), adult participants first completed a pretraining, in which each word was paired with two objects across trials: its target and another object (termed secondary target [T2]). Subsequently, participants learned the correct word‐object‐mappings in a supervised training paradigm and were then tested on the word meanings. During training, trials were structured such that some T2s never occurred with the targets, while others did, allowing us to disentangle the contributions of supervised and unsupervised pruning accounts. Eye movements were tracked during training and testing to measure the activation strength of alternative meanings. The experiments were identical but differed in how often the word was paired with the T2 during pretraining. We found that while weak incorrect associations were pruned quickly (Experiment 1), stronger ones remained present even after ceiling performance (Experiment 2), suggesting that the extent to which incorrect associations are unlearned depends on the strength of the initial mappings. Additionally, pruning was observed even for T2s that did not co‐occur with their corresponding word during training in line with unsupervised pruning. Overall, these findings imply that subtle incorrect associations may remain in the lexicon and contribute to other language processes (e.g., word recognition) even after word learning is completed.

## 1 Introduction

By adulthood, people typically know approximately 17,000 base words or word families (Goulden, Nation, & Read, 1990) and as many as 30,000 word forms (Brysbaert, Stevens, Mandera, & Keuleers, 2016). This is not a set of 30,000 independent pieces of knowledge: A single word form often maps onto multiple meanings (e.g., *bank* is both a financial institution and the side of a river), and a single meaning (e.g., a piece of living room furniture that seats multiple people) can map onto multiple word forms within the same language (i.e., synonyms: *couch*, *sofa*, *love seat*) or across different languages for a bilingual speaker (i.e., translation‐equivalent words: *divano* [Italian]). In addition, the lexical network does not just map auditory word forms to their meanings, they must also map word forms to articulatory movements and spellings. Thus, how people come to acquire this enormously complex network of knowledge is a critical scientific question.

One obvious property of a well‐organized lexicon is strong positive associations between to‐be‐connected word forms, meanings, and spellings. A less obvious property is that associations between words and *not*‐to‐be connected meanings are minimal (McMurray, Horst, & Samuelson, 2012). These incorrect associations could arise for a variety of reasons—they could be holdovers from an initial random state that must be pruned, or they could arise during learning as partially learned but incorrect mappings are briefly entertained. For instance, a child may erroneously learn that *spoon* maps to the concept of *broccoli* because broccoli frequently appears at the dinner table. However, incorrect associations could also be functional in some ways. For example, incorrect associations between *spoon* and the concept of a *fork* may partially capture the schematic or contextual relationships between them; or partial associations between *spoon* and the concept of a *ladle* may capture the fact that these are synonyms. Thus, the lexical network is likely not well‐described by a simple one‐to‐one mapping of words to concepts. Rather, there may be a variety of low‐level secondary associations that must also be managed.

The role and “management” of secondary associations is an understudied aspect of word learning. Thus, the present manuscript focuses on the secondary associations of the first type—the associations that are clearly incorrect and must be unlearned. Managing these associations may be essential for not only achieving the “correct” mappings, but also for achieving more efficient lexical processing (McMurray et al., 2012). Thus, learning a word may not only require “knowing” which meaning maps onto which word (strengthening correct connections), but also knowing which competitor meanings do not map onto that word (weakening incorrect connections). This study is motivated in part by the clarity of the framing (since it is clear that pruning must happen), but also by long‐standing debates between associative and hypothesis‐driven accounts of word learning (Medina, Snedeker, Trueswell, & Gleitman, 2011; Roembke & McMurray, 2016; Trueswell, Medina, Hafri, & Gleitman, 2013; Yu & Smith, 2007; Yurovsky & Frank, 2015), where the presence of these secondary associations at all is a clear source of evidence for a statistical or associative account over a hypothesis‐driven account.

Thus, the goal of this study is to set the stage for the investigation of this broader issue of managing secondary (or tertiary) associations by seeking to understand how learners minimize the strength and number of incorrect associations within the lexicon (e.g., how they are pruned). More specifically, we asked what type of information is necessary for incorrect associations to be weakened. Here, we distinguish between two different pruning accounts: supervised (e.g., unlearning of incorrect associations based on explicit feedback that an assumed word's meaning is not correct) and unsupervised (e.g., unlearning of incorrect associations based on a word's lack of a co‐occurrence with a specific meaning) as two distinct mechanisms for this pruning.

### 1.1 Word learning as an associative process

The contents of the adult lexicon are often conceptualized as an associative network linking words to other words and to sound, meaning, spelling, and other forms of knowledge (e.g., Collins & Loftus, 1975; Elman, 2004; McMurray et al., 2012). However, the idea that this network is the product of a gradual associative or statistical learning process—which would entail both strengthening and weakening connections—is more controversial.

A critical issue is the degree to which learners maintain multiple associations for a given word (which would entail the need for some form of pruning). Much research in *children's* word learning has been framed around the problem of referential ambiguity, the notion that every word learning situation is highly ambiguous due to the infinite number of potential referents for a given unknown word (Quine, 1960). Classic views motivated by this problem suggested that children require innate constraints and biases to guide word learning in these ambiguous situations (e.g., Markman, 1990). Although this view explicitly rejected an associative framing, it also did not make strong claims about whether children maintained one or more than one hypothesis for a single word.

In contrast, more recent statistical learning accounts suggest that children do not need to solve the problem of referential ambiguity at all. Rather, by gathering evidence across multiple naming events (“situations”), children can infer the correct mappings, even if they never solve the problem of referential ambiguity in any individual situation. These accounts—while still debated—have used an unsupervised statistical learning paradigm that offers clear evidence learners may track multiple hypothesized referents for a given word form. This line of thinking was started by Yu and Smith's finding of cross‐situational word learning (Smith & Yu, 2008; Yu & Smith, 2007).

In this paradigm, learners (infants, children, and adults) view a series of “situations” consisting of multiple potential referents. They then hear one or more target words. This perfectly captures Quine's framing—on any individual situation, there is no way to know what the word refers to. However, across multiple situations, only one object is consistently paired with a given word; thus, if learners track the relevant statistics, they can identify word‐referent‐mappings across trials, even in the absence of explicit feedback (i.e., supervised learning). This could be simply implemented via an associative model (McMurray et al., 2012; Yu & Smith, 2012), and crucially, it predicts that learners may have multiple potential candidates for each word, associated with various degrees.

However, a recent counterproposal is that learners could solve cross‐situational learning tasks without relying on associations if they maintain only one meaning for each word (a “hypothesis”) and test the accuracy of this hypothesis against novel situations (Medina et al., 2011; Trueswell et al., 2013). Critically, this approach—which is also unsupervised but leverages more powerful inferencing mechanisms—suggests that listeners should only have a single association between words and objects.

Much of the initial work contrasting these approaches focused on predictions of different models of trial‐by‐trial accuracy (Roembke & McMurray, 2016, 2021; Trueswell et al., 2013). However, a second line of work has more directly asked whether learners maintain more than one association or hypothesis with a given word.

A critical prediction of associative or statistical learning accounts is that when a word is paired with an object, associations between the two are strengthened; thus, over time, the associative strength between an object and a word should increase if they are paired consistently. However, this is conceived as a fairly general process—at any moment, all possible associations or statistical co‐occurrences can in principle be changed. Thus, the increase of associative strength between a word and an object is not limited to one mapping: Consequently, a word can also be associated with potentially incorrect references with low, subthreshold associations, even as this may not be evident in people's overt behavior.

For instance, a young learner may form an association between the word *couch* and the object *couch*, but may also possess a weaker, incorrect association between the word *couch* and the object *cushion* due to their frequent co‐occurrence in the living room.^1^ Similarly, for a second language learner of Italian, the object may also be less strongly associated with the word *divano*. Thus, the associative strength between a word and a number of semantic concepts is not static, but rather may be sensitive to the statistical surroundings of a word.

Yurovsky, Fricker, Yu, and Smith (2014) asked whether there is evidence for the gradual, partial accumulation of knowledge in word learning, as predicted by associative accounts. To do so, they first trained adult participants on a small number of word‐object‐mappings within a cross‐situational (unsupervised) word learning paradigm. Subsequently, they identified the words that had not been acquired (performance was at chance), and these words received additional training in a second learning session. The second phase also included a set of new words to use as a baseline. By comparing these new words to words that were not learned in the first training session, they asked if participants had learned any information about those words (e.g., via statistical co‐occurrence). Yurovsky et al. (2014) found that the words that had been newly introduced in the second phase were more easily acquired if they were paired with nonlearned words of the first phase. Thus, despite the absence of any measurable learning by the end of the first training phase, participants must have retained some partial knowledge about the original words. Yurovsky et al. (2014) data support the notion that people maintain subtle associations between words and objects, even if these connections do not necessarily result in readily observable accuracy benefits.

One way to measure these subthreshold associations more directly is to use a version of the visual world paradigm (VWP) to track participants’ eye movements as they complete a word learning task (Magnuson, Tanenhaus, Aslin, & Dahan, 2003). Here, the logic is that even as a person clicks on one object, they may be more likely to look at a competitor if it is also associated with the word they heard. This captures partial activation for latent associations and provides clear evidence for the parallel consideration of correct and incorrect associations on the same trial if the participant is simultaneously looking at a competitor but clicking on the target.

To test this, Roembke and McMurray (2016) trained participants on eight word‐object‐mappings in a cross‐situational word learning experiment. Each word was always paired with its target object across trials, and foil objects were pseudo‐randomized. However, at the same time, each word was also paired with a high‐co‐occurrence (HC) competitor on 60% of trials, and with a low‐co‐occurrence (LC) competitor (40% of trials). Unrelated foil objects co‐occurred with a word at approximately 20%. Over the course of training, participants were more likely to look at the HC competitor than a randomly chosen foil. Importantly, this was even the case as they simultaneously clicked the correct object, indicating that participants maintained multiple word‐object‐mappings at the same time. Moreover, there was some limited evidence that the relative amount of looks to the HC competitor increased with time (Roembke & McMurray, 2016); this suggests that these secondary associations are enhanced as learners encounter the word with the HC competitor. This suggests that the unsupervised co‐occurrence statistics of words and objects can strengthen specific word‐object‐mappings.

While there is an ongoing debate around the exact nature of the underlying mechanism of word learning (e.g., Berens, Horst, & Bird, 2018), these data from converging paradigms suggest that associative learning is at least partially responsible and provide clear evidence that people can form multiple partial associations for a given new word. Consequently, during learning, as correct associations are built over time, it may be unavoidable that spurious associations occur as well. This raises the question of how these associations are managed or suppressed to get to a more efficient and accurate network.

### 1.2 Negative associative learning

While the discussion so far has focused on work on human learning, the strong grounding in associative models suggests the need to consider broader work—including computational models and nonhumans—on associative learning.

Converging evidence from a number of broader domains suggests an important role for pruning over development, although it is unclear if this relates to many‐to‐many learning. For example, neural pruning of unneeded dendritic branches or even neurons is well‐known throughout development (Huttenlocher, 1979), children are known to eliminate inefficient mathematical numerical strategies as they acquire numerical competence (Siegler, 1989), and rodent prune motor patterns during sleep (Blumberg, Coleman, Gerth, & McMurray, 2013). However, these are broader developmental changes—not specific learning paradigms.

Computational modeling also posits a strong role for negative associative learning. Many connectionist models, for example, start with all weights initialized to small random values; as most weights are ultimately not needed, many of these later need to be suppressed during learning. For example, McMurray et al. (2012) developed an (unsupervised) connectionist model in which networks learned to map word forms onto visual referents. In their model, gradual associative learning was quasi‐independent of real‐time processes such as the ability of the network to identify the referent of a word in the moment. This approach allowed the model to simulate developmental phenomena like the ability to fast‐map a novel word onto an unknown object. Importantly, the model suggested that the pruning of incorrect associations may play an important role in accounting for lexical development: For example, how quickly a word was activated was more dependent on how well‐pruned incorrect associations were, in contrast to how strong the connection between the correct object and word was. Similarly, the model's ability to fast‐map a novel term onto a novel object was dependent on which spurious connections between objects and words had been pruned or not (McMurray et al., 2012).

Despite the importance of these connections in modeling projects, direct empirical evidence for a role of negative associative learning is missing. Lab‐based studies of word learning are often limited to teaching adults small vocabularies of nonwords. As a result, it can be difficult to isolate any purely associative components to learning because adult humans can engage in higher‐level, inferential mechanisms to accelerate learning. In contrast, word learning experiments in young children often include ostensive teaching of a small number of words. For example, a child is presented with a single object that is repeatedly labeled (e.g., “Look, it's a *toma*! That's the *toma*”) in the absence of any object competitors (e.g., Ferguson, Havy, & Waxman, 2015; Namy, Campbell, & Tomasello, 2004; Woodward & Hoyne, 1999).

One way to distill the associative mechanisms to a more tractable form is to consider work on associative learning in largely animal models (where such strategies are less likely). Here, it is important to note that the classic (largely animal‐based) work on associative learning differs markedly from the version of associative learning posited for human word learning, as the former is supervised (requiring explicit feedback on the response), while the latter is unsupervised. This is in part out of necessity—animals will do very little without reinforcement, even as the child's language learning environment is thought to feature little feedback. This is an important distinction that we return to momentarily.

In the animal learning literature, associative learning has long been hypothesized to include both positive (the strengthening of correct associations) and negative (the pruning of incorrect associations) forms (Hearst, Besley, & Farthing, 1970; Rescorla & Wagner, 1972; Spence, 1937; Thorndike, 1898). Nevertheless, most research has been concerned with how correct associations are built over time (Mackintosh, 1974; Newport, Wallis, Temple, & Siebeck, 2013; Stout & Miller, 2007), leaving unclear how critical the elimination or pruning of incorrect associations is for learning. Importantly, many classic studies of associative learning do not train a set of mappings that is complex enough to capture anything similar to word learning in which a large number of items (words) must be mapped to a large number of responses (referents), a many‐to‐many mapping.

Thus, to investigate associative processes during vocabulary acquisition in a many‐to‐many learning paradigm, Roembke, Wasserman, and McMurray (2016) adapted an animal model of word learning for pigeons (developed by Wasserman, Brooks, & McMurray, 2015). In this task, pigeons were trained on 16 symbol‐object‐mappings in a supervised learning paradigm, where they received feedback on each trial. The pigeons were trained in a 2AFC task in which they saw a single real object (e.g., a car) and had a choice of two symbols on a touch/peck screen: one was the target, and one was a randomly selected foil. They received feedback after pecking one of the symbols. It was assumed that during training, they would strengthen the associations between the object and the correct symbol and weaken associations between that object and the incorrect symbol.

To investigate negative associative learning, mappings were separated into two clusters, so that the foil (the incorrect response) for a trial always came from the set of eight items that were in the same cluster as the target object. This manipulation guaranteed that there would be a set of symbols that consistently appeared with any given target (those in the same cluster) and a set that never co‐occurred with that target (the symbols in the *opposite* cluster of the target), even as they were familiar symbols (since they were targets and foils on other trials).

Supervised accounts of associative learning posit specifically that associations are pruned when the learner chooses that option and receives negative reinforcement. Under this view, the associations between the target picture and the competitors in the opposite cluster could not undergo negative associative learning, as they never co‐occurred with the target and, therefore, could not have been chosen by mistake to then receive negative reinforcement.

After training, pigeons were then tested on trials in which the foil symbol came from the same cluster as the target, or was selected from the opposite, unpruned cluster. Pigeons’ accuracy was lower on trials with the foil in the opposite cluster. If learning these mappings had only depended on how strong the association between a target symbol and an object were, pigeons should have performed equally well in both training and testing trials. Instead, the higher, never‐pruned associations to the foil disrupted their performance. Pigeons were also tested on trials in which the target symbol was not present (so‐called “no‐correct” testing trials). Here, the responses consisted of an *in‐cluster* (pruned) symbol and an *out‐cluster* (unpruned) symbol. In no‐correct testing trials, pigeons were more likely to select the *out‐cluster* foil than the *in‐cluster* one. If incorrect associations had not been pruned, pigeons should have selected the *in‐cluster* symbol, which had co‐occurred with the object during training.

These results suggest that some form of negative associative learning is operative when acquiring many‐to‐many symbol‐object‐mappings: That is, associations with the foils from the same cluster were pruned despite receiving continued unsupervised support due to the foils’ co‐occurrence with the target symbol during training. At the same time, associations with foils that only occurred in the opposite cluster were not pruned, even though they never co‐occurred with the target symbol and were actually consistently paired with an alternative symbol. Thus, at least in an animal model, these results indicate that supervised statistics (i.e., negative feedback) are more critical for such pruning than unsupervised ones (e.g., co‐occurrence).

There are clear differences between this animal model and human word learning. As argued by Wasserman et al. (2015), studying pigeons can isolate associative learning from higher‐level executive processes. However, this raises the question of how critical negative associative learning is in more complex, cognitively rich animals like humans. It is possible that humans use executive functioning or strategies to quickly overcome any spurious associations that may have formed or to avoid forming them in the first place. Even though this process would also include pruning at a functional level, the mechanism at work may be very different.

Perhaps more importantly, the transition to humans raises the possibility that unsupervised learning is occurring simultaneously with supervised learning. That is, even as this was conceived as a supervised learning paradigm (as is normal in the animal learning literature), the kinds of unsupervised statistics posited by Yu and Smith (2007) are still present. Under this view, referents that never co‐occur with the target word should be pruned (since they have no co‐occurrence probability). In the context of the Roembke et al. (2016) study, the *out‐cluster* foils actually have less co‐occurrence with the target picture than the *in‐cluster* foils and so should have been more thoroughly pruned. This is not what was observed in pigeons (the *in‐cluster* foils were responded to below chance). However, in humans, where unsupervised learning is more thoroughly attested, a design using the clustered foils raises the possibility to pit supervised versus unsupervised mechanisms of negative associative learning.

### 1.3 The present study

**Fig. 1:**
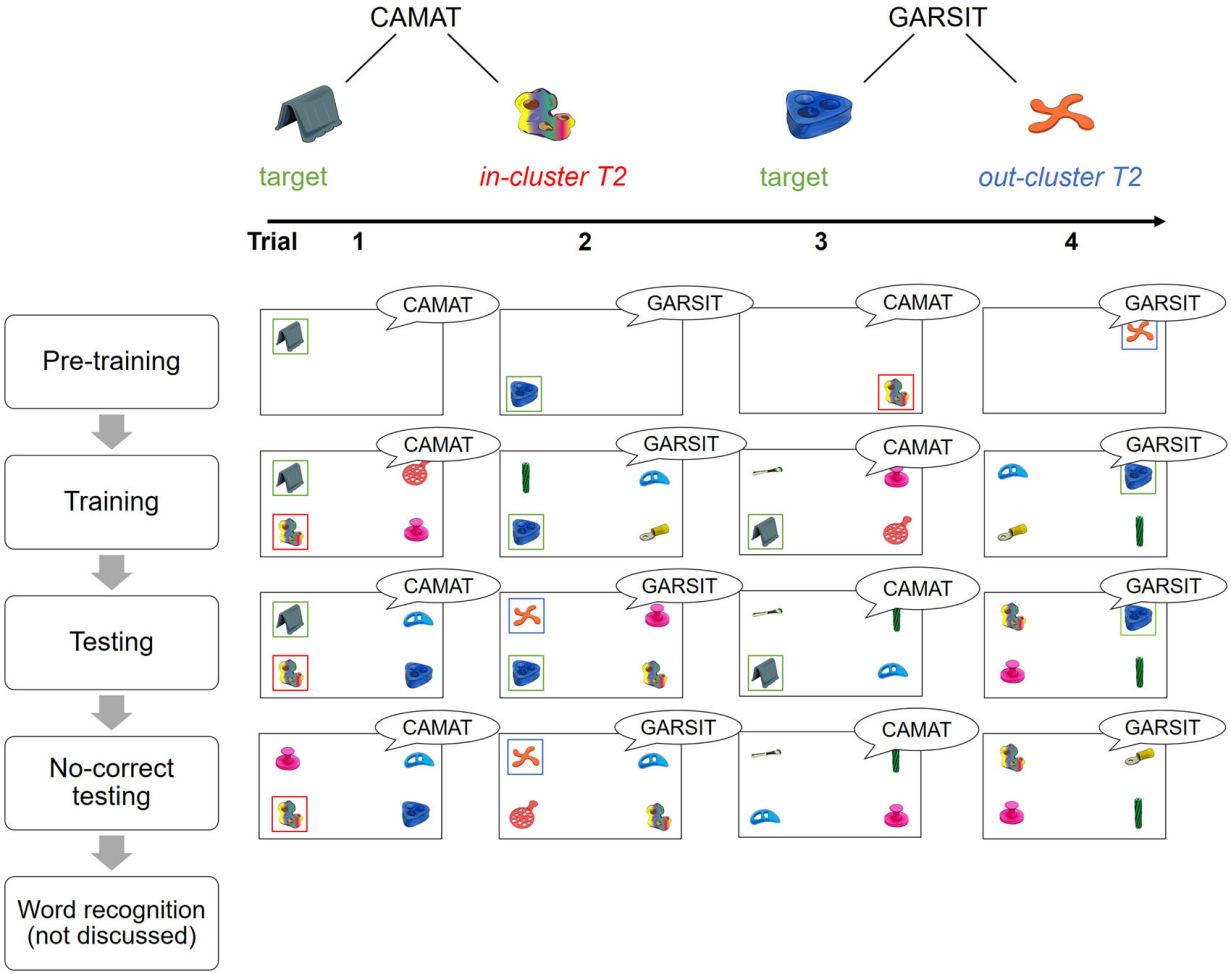
Overview of the basic structure of both experiments. *Note*. Top row: Words are linked to two objects during pretraining, a target object (T1) (highlighted in red) and a secondary object (T2; in green or blue to denote *in‐cluster* or *out‐cluster* status). *In‐cluster* objects are objects that co‐occur with the word during training, while *out‐cluster* objects are objects that do not co‐occur with the word during training. Bottom rows: One screen represents one trial and each row represents a phase of the experiment. Frames around the objects are added to indicate whether the object was a primary target (green), secondary target T2 (red/blue), or a foil (not outlined). Frames were not present during the actual experiments. Pretraining taught the initial associations of the word with the target and T2s. During training, the target was always present; *in‐cluster* T2s (marked red) could appear with their label (e.g., *camat*; Trial 1 of training). Though *out‐cluster* T2s (marked blue) were treated as incorrect, they could not co‐occur with their label (e.g., *garsit*) during training. During testing, both *in‐cluster* and *out‐cluster* T2s occurred with their label (e.g., Trials 1‐2 of testing). During no‐correct testing, target objects never occurred with their label, while *in‐cluster* and *out‐cluster* secondary targets could (e.g., Trials 1‐2 of no‐correct testing). Eye movements were tracked from the training session onward. Data from the word recognition section are not reported here.

The present study asked whether connections between words and objects are not just strengthened during learning, but also pruned. For this purpose, two experiments were conducted, in which each word was first associated with both a target object (abbreviated as T1) and a secondary target (abbreviated as T2). We then asked how the association with T2 was *unlearned* over time. Each experiment consisted of several phases (see Fig. 1 for an overview). (1) Pretraining used a simple word/object association task to associate the word with both its target and T2; (2) training used a supervised learning paradigm to further strengthen the associations and potentially prune the T2 association; (3) testing; and (4) no‐correct testing used different designs to probe the strength of these associations.^2^ The design of the two experiments was identical with the exception that Experiment 2 built a stronger incorrect association during pretraining: In Experiment 1, an object co‐occurred more frequently with its target than its T2 during pretraining, simulating a weaker, secondary association. In Experiment 2, in contrast, an object co‐occurred more with its T2 than its eventual target during pretraining, simulating what is essentially a change in the meaning of a word.

To facilitate understanding of the experimental design, we first describe the different experimental phases in more detail. Subsequently, we outline the clustering design that allowed us to pit supervised and unsupervised pruning accounts against each other (similarly to what was done in pigeons; Roembke et al., 2016). Finally, we will state hypotheses for each experimental phase, based on the two accounts of pruning.

#### 1.3.1 Description of the different experiment phases

Each experiment started with a pretraining phase in which adults were deliberately trained on a mapping between the word and two objects in isolation (similar to Vouloumanos, 2008). On each trial, only a single object and novel word were presented. However, across trials, each word was associated with two distinct objects: its target and what we termed the T2. In Experiment 1, T2 was slightly less frequent than the target (T1: 60% / T2: 40%), while this was reversed in Experiment 2 (T1: 20% / T2: 80%). Thus, by the end of pretraining, two associations were established for a given word, one stronger than the other. This then enabled us to ask how and if they were lost in the subsequent training.

After this initial pretraining, participants completed a more traditional supervised learning paradigm loosely modeled after Roembke et al. (2016) prior work with pigeons. On each trial, they heard one word with four potential objects on the screen (the target and three foils). On some trials, one of these foils was the T2 that had been reinforced during pretraining. Participants then responded and received feedback based on the object they selected. Critically, if learners were using negative reinforcement to prune the T2 connections, we expected that it was on these trials that they may “erroneously” choose the T2, get reinforced, and then prune their connections. The procedure of the subsequent testing session was identical to training but did not include feedback.

During training and test, we tracked eye movements to potential alternative interpretations using a variant of the VWP (Magnuson et al., 2003; Roembke & McMurray, 2016). We examined fixations to competing objects on only trials where participants eventually selected the correct target. Following Roembke and McMurray (2016), this constitutes strong evidence for parallel activation of both the target and competitor, as people are simultaneously (on the same trial) clicking the target but maintaining above baseline fixations to a competitor.

Finally, an additional short test session was conducted, in which the target object was not included as one of the four presented options (no‐correct testing trials; following Roembke et al., 2016) work with pigeons). Half of the trials included a word's T2. Here, the logic was that without the target present, learners would be at chance. If they had pruned the T2, they may then be less likely to choose it than a foil competitor.

#### 1.3.2 Clustered design

**Fig. 2:**
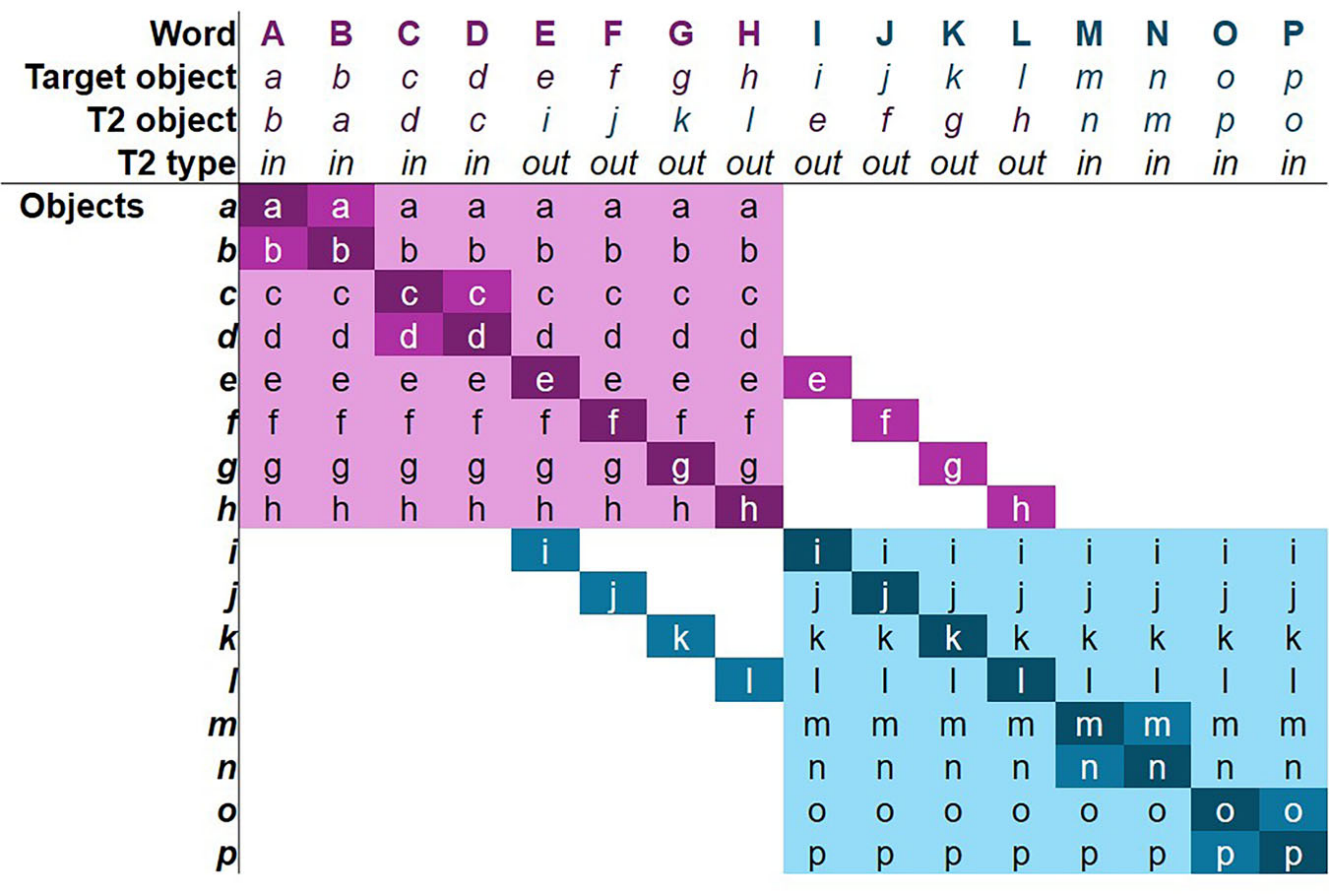
Overview of clustering design. *Note*. The table shows the possible foil objects during training (rows) for each word (columns). Words and objects are denoted by capital and lower case letters as the precise assignment of words and objects to roles in the design was randomized by subject (e.g., for some participants, word A was *turgon* and object a was a gray tent‐shaped thing; but for other participants, word A was *camat* and object a was the green spiral. The darkest cells indicate the target object (e.g., Word A with object a), intermediate cells are the secondary target, T2. Light‐colored cells are possible foils. Note that *out‐cluster T2s* are shown (e.g., word E with object i). These did not co‐occur with the target word during training but could occur at test.

The key manipulation in this study was that during training, targets and foils were clustered, using a similar design to Roembke et al. (2016). Words and targets were separated into two clusters (see Fig. 2), where half of the word‐object‐mappings were part of one cluster and the other half part of another. During training, foil objects could only come from within the same cluster as the target object. The result of this is that from a supervised learning perspective, some foils will receive opportunities for negative associative learning (the foils in the cluster for the target word), whereas others did not (since they could not serve as responses). For example, based on Fig. 2, foil objects for Word A could only be Objects b–h.

**Fig. 3:**
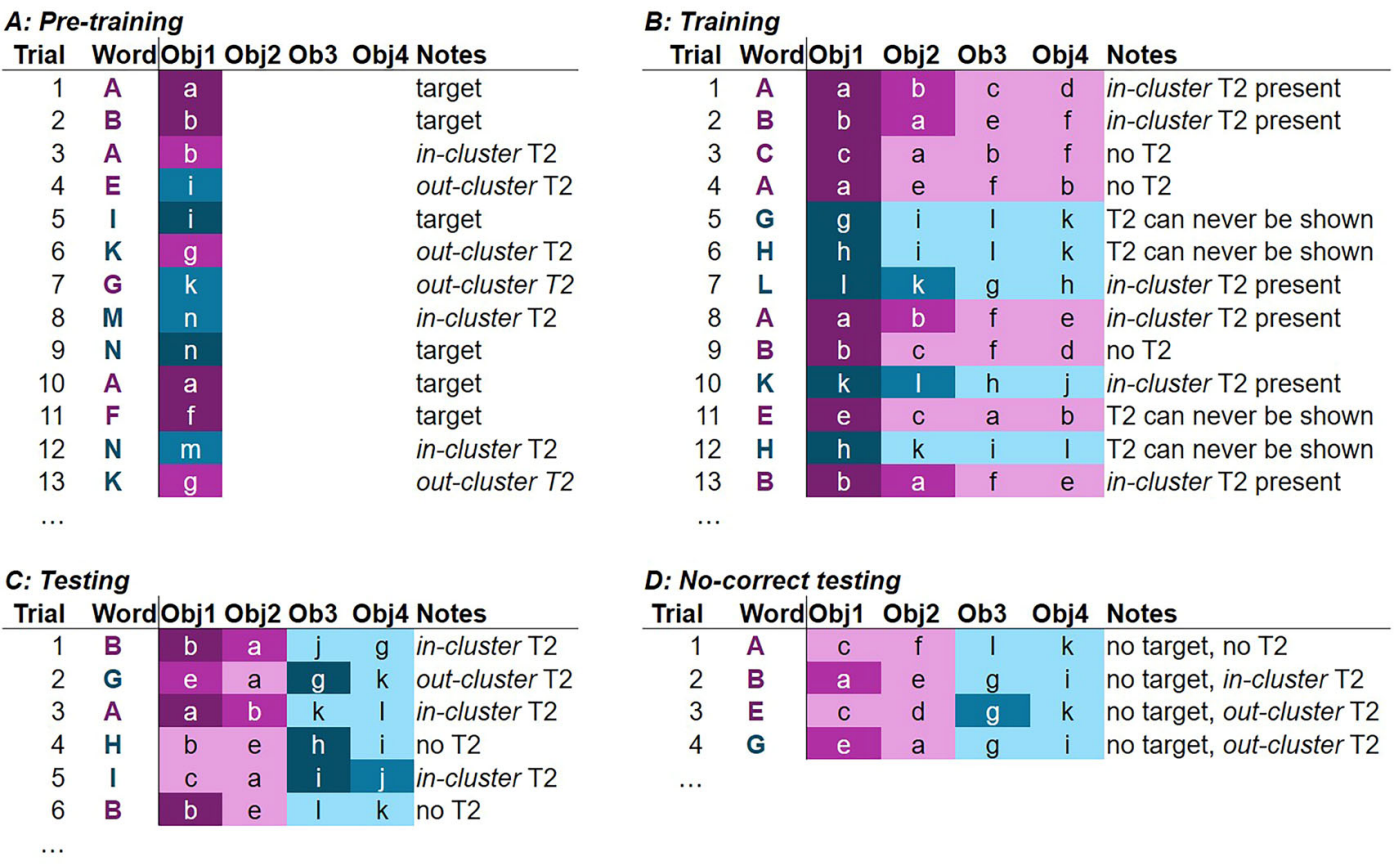
Sample sequence of trials linked to the clustering design. *Note*. Color coding is the same as in Fig. 2. Each row is a trial, with the possible responses/objects shown in separate columns. Objects are arranged in “order” (target first, then the secondary target [T2], by cluster if applicable), but in the actual experiment, they were randomized on the screen. (A) During pretraining, each word appeared with a single object on each trial, either the target or its T2. *Out‐cluster T2s* will appear as a mismatch between the color of the word and the object. (B) During training, words always appeared with the target; for words with an *in‐cluster T2*, the T2 could appear alongside the other foils; for words with an *out‐cluster T2*, only foils were shown. (C) At test, each trial would have two objects from each cluster, a target and sometimes the T2. (D) During no‐correct testing, the target was not shown. Some trials had all foils; others would include the T2.

Fig. 3 shows how the clustering and the assignment of secondary targets T2 would play out across a set of trials in each phase of the experiment. This clustering interacted with the T2s (from pretraining) in systematic ways. For half of the words, the T2 belonged to the same cluster during training (e.g., Object b for Word A); it is termed the *in‐cluster T2*. For the other half of the words, the T2 was assigned to the opposite cluster (e.g., Object i for Word E). This is referred to as the *out‐cluster T2*. Because foils were always chosen from the same cluster as the target word, this meant that across training trials (Fig. 3B), an *in‐cluster T2* would appear from time to time, but *out‐cluster T2s* would never appear. Consequently, from a supervised learning perspective, we would expect the *in‐cluster T2s* to undergo substantial pruning, while the *out‐cluster* T2s could not (since the T2 was never presented alongside the word). In contrast, from an unsupervised learning perspective, the *out‐cluster T2s never* co‐occurred with the target word (after pretraining) and, therefore, would have undergone *more* pruning.

During training, the two clusters never overlapped within the same trial. However, this was no longer true at test (Fig. 3C). Here, a T2 was present on a subset of trials (along with a baseline foil); hence, the competing object could now consist of either the *in‐cluster* or *out‐cluster T2* (depending on the target word) or a baseline foil. This allowed us to use the VWP to ask whether pruning of incorrect associations was differentially stronger for the *in‐cluster T2* (which would have had the opportunity for negative associative learning during training) than the *out‐cluster* T2 (which did not). Similarly, during no‐correct testing (Fig. 3D), trials were clustered in a way that some included the T2 or not. Across trials, we asked if participants were more likely to select the *out‐cluster T2* (i.e., an unpruned competitor) over a specific foil (as in the pigeons in Roembke et al., 2016).

#### 1.3.3 Predictions

**Fig. 4:**
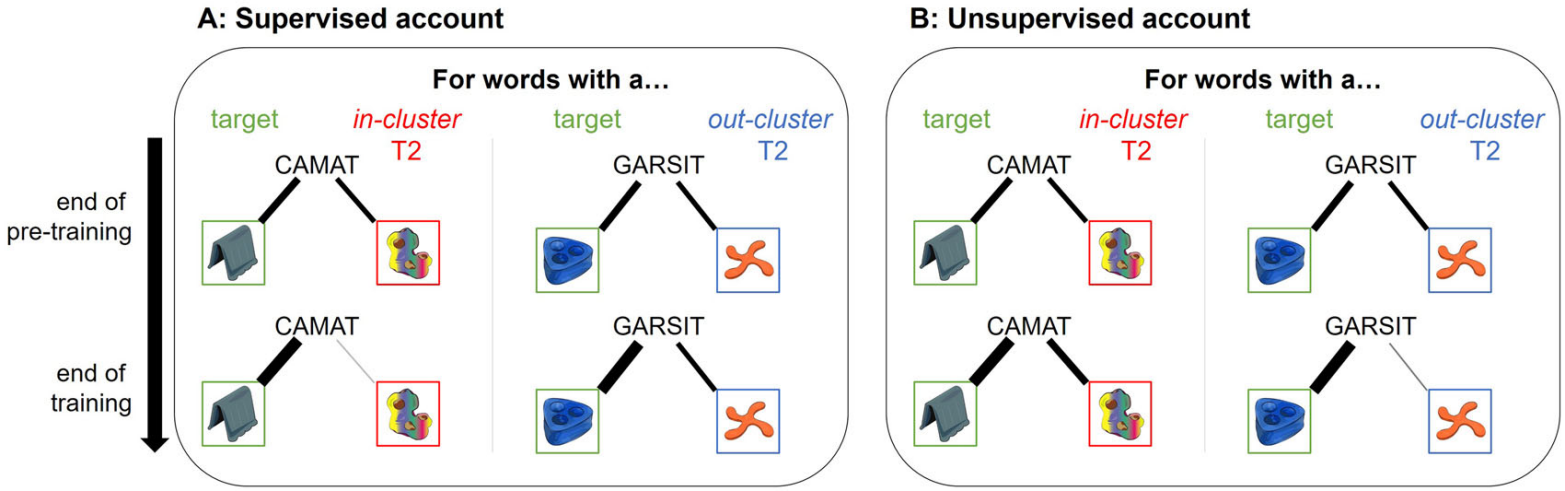
Overview of the predictions of a supervised (Panel A) and an unsupervised (Panel B) account of pruning. *Note*. The figure is consistent with Experiment 1's design, where each word was roughly equally likely to be presented with its target and secondary target. The thickness of the lines between the words and objects indicates the strength of their associations, and how these associations are predicted to change over the course of the experiment. The strength of association is thought to be reflected in the number of eye movements participants make to an object. For example, a supervised account predicts that associations between a word and its *in‐cluster* T2 will become weaker (due to pruning), resulting in a lower number of eye movements to the *in‐cluster* T2 by the end of training.

We predicted that participants would form associations between words and objects based on the co‐occurrence statistics that they encountered during pretraining (Vouloumanos, 2008). That is, we expected that objects would be relatively equally associated with their target and T2 after Experiment 1's pretraining. In contrast, for Experiment 2, we predicted that objects would be more strongly associated with their T2 than their target (see Fig. 4).

During training, we predicted that participants’ looks to the *in‐cluster T2* would be higher than to baseline foils at the beginning (since these had been reinforced during pretraining). Consistent with the difference in pretraining co‐occurrence statistics across experiments, we predicted that looks to the *in‐cluster T2* would be more pronounced during Experiment 2 than in Experiment 1.

A *supervised account of pruning* then predicts looks to the *in‐cluster T2* should decrease over the course of training as incorrect associations were pruned. That is, for *in‐cluster T2s*, these objects could appear as foils during training and would occasionally be selected. Consequently, supervised learning could use negative feedback to prune these incorrect associations from pretraining (Fig. 4A), and even positive feedback from a correct response would lead to some pruning of the available responses (e.g., the *in‐cluster T2*).

Our primary test of our hypothesis was at test where we could contrast accounts of pruning. As stated, during training, there was ample opportunity for subjects to prune the *in‐cluster T2* via reinforcement, thus at test a *supervised* account of pruning predicts they should receive few fixations. In contrast, for an *out‐cluster T2*, under a supervised pruning account, there would be *no* opportunity to prune incorrect associations with the word, as they never co‐occurred during training and, therefore, never received negative feedback. Thus, they should receive substantial fixations.

In contrast, an *unsupervised account of learning* makes the opposite predictions (Fig. 4B). During training, the *in‐cluster T2* co‐occurred frequently with the target word. This could result in participants' maintaining their association between words and their *in‐cluster T2*, resulting in significant fixations above baseline. However, the *out‐cluster T2s* would have a co‐occurrence of 0 with the target, and thus their associations could be pruned that way. Finally, for no‐correct testing, a supervised account predicts they would select the *out‐cluster T2* at greater than chance levels, but the *in‐cluster T2* would be selected at chance level. The unsupervised account predicts the converse.

Of course, it is possible (and even likely) that both forms of pruning are operative. In this case, we might predict no difference between *in‐* and *out‐cluster T2s*, but the degree to which both get more fixations than the foils might indicate whether pruning happened at all.

## 2 Experiment 1

### 2.1 Method

#### 2.1.1 Participants

Participants were 40 monolingual native English speakers with normal or corrected‐to‐normal vision (30 females, 10 males, 0 nonbinary/preferred not to answer); all were adult students at the University of Iowa and received course credit for participation. No age information was collected for participants. One participant did not complete the whole experiment due to time restrictions; their data were included for the phases they did complete. Participants underwent informed consent as part of an IRB‐approved protocol.

#### 2.1.2 Stimuli

Referents were photographs of unusual objects that had been converted to clipart images. Words were two‐syllable nonwords (Table 1). These were created to have a strong real word onset competitor for an exploratory analysis of word recognition which was conducted at the end of the experiment (and not reported in this manuscript). None of the nonword targets were competitors of each other. Auditory stimuli were recorded by a female, native speaker of English in a neutral carrier phrase (e.g., “He said…”). Subsequently, five exemplars of each stimulus were selected and edited to remove extraneous elements that were not part of the stimulus (e.g., jaw clicks). Finally, auditory stimuli were normalized and 50 ms of silence was added to their beginning and end.

**Table 1:** Novel words used in Experiments 1 and 2

Written form	IPA
beamler	/bimlɚ/
camat	/kæmæt/
dellet	/delet/
fumlic	/fumlɪk/
garsit	/gærsɪt/
hustrim	/hʌstrɪm/
jospit	/tʃɔːspɪt/
lindle	/lɪndle/
mougger	/maʊgɚ/
nocket	/nɔket/
parshim	/pærʃɪm/
raimmer	/reɪmɚ/
sauble	/sɑble/
shemster	/ʃemztɚ/
turgon	/tɝgɔn/
wammock	/wæmɔk/

#### 2.1.3 Design

Participants learned 16 word‐object mappings. To create word‐object‐pairings, we first assigned one target object to each word randomly for each participant. Subsequently, words were paired in four possible ways, where the target of one word became the T2 of the other and vice versa. Half of the words were assigned to Cluster A and half to Cluster B in a way that half of the word pairs that shared target/T2s were in the same cluster and half were not.

Participants first completed the pretraining. This was followed by three blocks^3^ of training, a testing session, and the no‐correct testing. Last, participants completed a word recognition phase to examine how novel words competed with known words (see Fig. 1 for a schematic). The experiment overall took approximately 1.5 h.

Pretraining consisted of 160 trials (10 trials/word) during which only one object was presented on each trial. Each word was paired with its target object on 60% of all trials and its T2 on 40% of all trials. The object was randomly presented in one of the four corners to encourage participants to pay attention to the object that was paired with each word. The order of trials was random during pretraining.

During training, participants heard one word and saw four objects, one of which was always the target. Training consisted of three blocks of 112 trials (7 trials/word; 336 trials overall). Within each block, three out of the seven trials per word always included the T2. This was the same co‐occurrence rate as any other object from the same cluster so that the co‐occurrence of word and T2 (within this phase) was not different from other objects during training. Thus, any difference between T2s and other object competitors must be due to the exposure during pretraining. Participants received feedback on their responses during training.

Testing consisted of one block of 160 trials (10 trials/word). Testing trials consisted of several trial types. For all trial types, two objects came from the same cluster as the word, and two came from the other cluster. Thus, participants could not guess what word they would hear based on the distribution of response options (e.g., if three objects were from the same cluster, this may bias participants to select a response option from the over‐ or underrepresented cluster). *In‐cluster T2* testing trials included the target object, the *in‐cluster T2*, and two baseline foils that were randomly chosen from the other cluster (out‐foils). *Out‐cluster T2* testing trials included the target object, the *out‐cluster T2*, and two baseline foils, one of which was from the same (in‐foil) and one of which was from the other cluster (out‐foil). Control testing trials included the target object and three baseline foils (two out‐foils and one in‐foil). There were three *in‐cluster* or *out‐cluster T2* testing trials per word during testing and seven control testing trials per word. All foils (except for the T2) were randomly chosen without replacement from the respective cluster.

Finally, participants completed a no‐correct testing session of 32 trials (2 trials/word). During this session, trials never included the target object as a response option. Instead, four foil objects were presented as possible responses, two from each cluster. For each word, one trial included the T2. For trials including the *in‐cluster T2*, baseline foils were one *in‐cluster* foil and two *out‐cluster* foils. For trials including the *out‐cluster T2*, baseline foils were two *in‐cluster* foils and one *out‐cluster* foil to complete the set. The remaining trials for each word always included two baseline foils from each cluster. In the no‐correct session, one of the two trials for each word was assigned to either the first (trials 1–16) or the second half (trials 17–32). The order of trials was randomized within those halves. Participants did not receive feedback during the no‐correct session.

Trials were always randomized within block, unless otherwise noted. The location of each object was randomized across the four possible locations on each trial.

#### 2.1.4 Procedure

The experiment was conducted in a sound‐attenuated room. The display was a 19ʼʼ monitor operating at 1280 × 1024 resolution, and sounds were presented via high‐quality headphones at a volume comfortable to the subject.

Participants were informed that they would first complete a pretraining with only one object on each trial, before advancing to a training session with feedback, followed by a testing session without any feedback.

For pretraining, they were told the following: “In this study, you will be learning what objects a few new words identify. First, you will complete a pre‐training. Each trial begins with a blue dot in the center of the screen. When the dot has turned red, click on it with the mouse. Then, you will hear a word. After hearing the word, click on the present object that matches the word. When you are ready to begin, press the space bar.” During pretraining, participants were presented with a small blue circle at the center of the screen. In addition, one object appeared in one of the four corners of the screen. Participants were given 1050 ms to inspect the object. Afterward, the circle turned red, cueing the participant to click on it to hear the word. When the participant clicked on it, the red circle disappeared, and the target word was played. The auditory stimulus was randomly selected to be one of the five exemplars of the target stimulus (with replacement). To move on to the next trial, participants had to click on the presented object.

For training, participants were given the following instructions: “You will now see on each trial four objects and hear a novel word. Your job is to try to determine which object goes with which word. After hearing the word, click on the object that matches the word. In the beginning, you will need to guess, but your responses should become more informed over time.” Participants were also told that they would receive feedback, indicating whether they clicked on the correct object or not. Before starting training, participants then heard the two types of sounds that would be used for feedback.

During training trials, participants saw a small blue circle at the screen center and four objects in the corners. As before, the blue circle turned red after 1050 ms, and the auditory stimulus was played when participants clicked on it. Participants then clicked on the picture that they thought corresponded to the word. Finally, they received feedback indicating if their response had been correct or incorrect. Feedback consisted of either a high tone (“Bing!”), indicating a correct response, or a low tone (“Mep!”) for an incorrect one. The display turned white when participants responded and stayed so until the end of feedback; feedback was played after approximately 150 ms. Trials were not time‐limited.

Testing and no‐correct trials were identical to training trials with the exception that participants did not receive any feedback. For testing, they were then told that they would complete a block of testing without feedback. Finally, for no‐correct testing, participants were explicitly told that trials would not include the target object: “You will complete another short block. This time, the trials will NEVER include the target. Make a best guess what the word could mean. You will never receive feedback.”

#### 2.1.5 Eye‐tracking recording and analysis

Eye movements were recorded using an SR Research Eyelink 1000 chinrest mount eye‐tracker operating at 250 Hz. No eye movements were recorded during pretraining (with only one object present during pretraining, eye movements were not meaningful). Both corneal reflection and pupil were used to obtain a point of gaze whenever possible, though, for some participants, only good pupil readings could be obtained.

At the start of the experiment, participants were calibrated with the standard 9‐point display. Every 30 trials, a drift correction procedure was conducted to check adequate calibration and account for any drift in the track. Preprocessing was conducted using the EyelinkAnalysis framework (McMurray, 2023). Fixations were automatically parsed into saccades and fixations using the default “cognitive” parameter set. Adjacent saccades and fixations were combined into a single “look” (starting at the onset of the saccade and ending at the offset of the fixation as in prior studies; McMurray, Aslin, Tanenhaus, Spivey, & Subik, 2008, 2010). To account for noise in the eye‐tracking record, the ports containing the objects were extended by 100 pixels when computing the point of gaze. No overlap between the objects resulted from this.

The dependent variable in eye‐movement analyses was the “area under the curve” between 300 and 2000 ms. We started by computing the standard fixation curve which computes the proportion of trials on which the participant was fixating a given competitor at each moment in time (for each object). Next, we averaged that between 300 and 2000 ms to get the average looking to that object (in that condition). The lower limit of 300 ms was selected because people typically take approximately 200–250 ms to launch an eye movement. Thus, considering both the delay for oculomotor planning and the 50 ms silence at the beginning of each word, any eye movements before the lower boundary of 300 ms likely were not driven by the word they heard. The upper limit of 2000 ms was the default end of the analysis window (in EyelinkAnalysis) and is always used by our lab for studies of typical adults.

#### 2.1.6 General statistical methods

Data were analyzed using linear mixed‐effects models in R (version: Ri386 4.1.2; R Core Team, 2020), using the *lmer* function. We used the lme4 (version 1.1‐31), nlme (version 3.1‐160), and lmerTest (version 3.1‐3) packages for all analyses. The lmerTest package was used to estimate degrees of freedoms using the Satterwaithe approximation and compute *p*‐values from the mixed models. A *p*‐value below .05 was deemed significant.

Possible random effects included subject, word, and target object. The simplest model always included the random intercept of subject. To find the most complex random effects structure needed for the data (Matuschek, Kliegl, Vasishth, Baayen, & Bates, 2017), we used a chi‐square model comparison test to evaluate nested models and we report chi‐square statistics for the comparison of the most complex model that reached significance and the less complex one before, unless no additional random intercepts and slopes were required.

### 2.2 Results

#### 2.2.1 Training

**Fig. 5:**
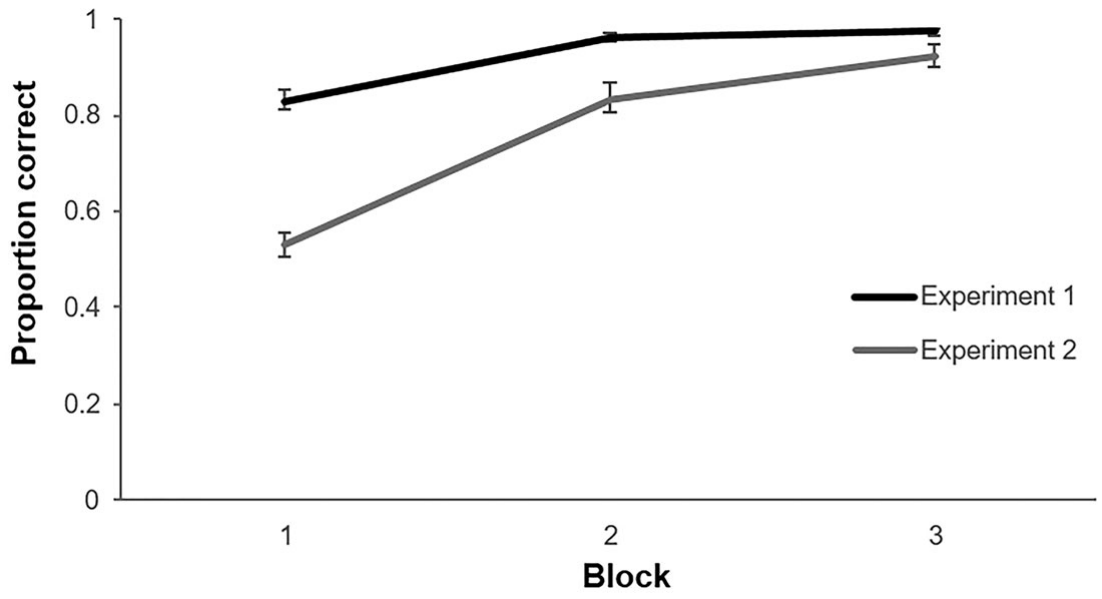
Proportion correct during training blocks of Experiments 1 and 2. Error bars indicate the standard error of the mean.

Accuracy was very high: Participants performed above 80% correct by Block 1 (trials 1–112) and near ceiling by Block 3 (Fig. 5). We first investigated participants’ response choices during Block 1 of training, to determine if participants showed evidence of having acquired the pretraining statistics. To do this, we specifically looked at words where the T2 was available as a response option. For these, participants selected the target on 79% of the trials (for comparison, in control trials, this was 84%), the *in‐cluster T2* on 12% of the trials, and one of the baseline foils on 6% (average of the two baseline foils) of the trials. Response selections were converted to log‐odds ratios and analyzed using a one‐sample *t*‐test. The difference between *in‐cluster T2* and baseline responses was significant, indicating that participants must have encoded the incorrect association between a word and its T2 during pretraining (*t*(39) = 3.91, *p* < .001). In Blocks 2 and 3, the *in‐cluster T2* was selected on 3% and 2% of possible trials, respectively. This is a slightly higher selection rate than the one of the foils (1% in Blocks 2 and 3), though, this was not significant (*p* > .05). Nonetheless, participants were heavily biased to the target (over the *in‐cluster T2*) in even the first block, suggesting the extra association with the *in‐cluster T2* was quickly eliminated—at least as indicated by the overt responding.

**Fig. 6:**
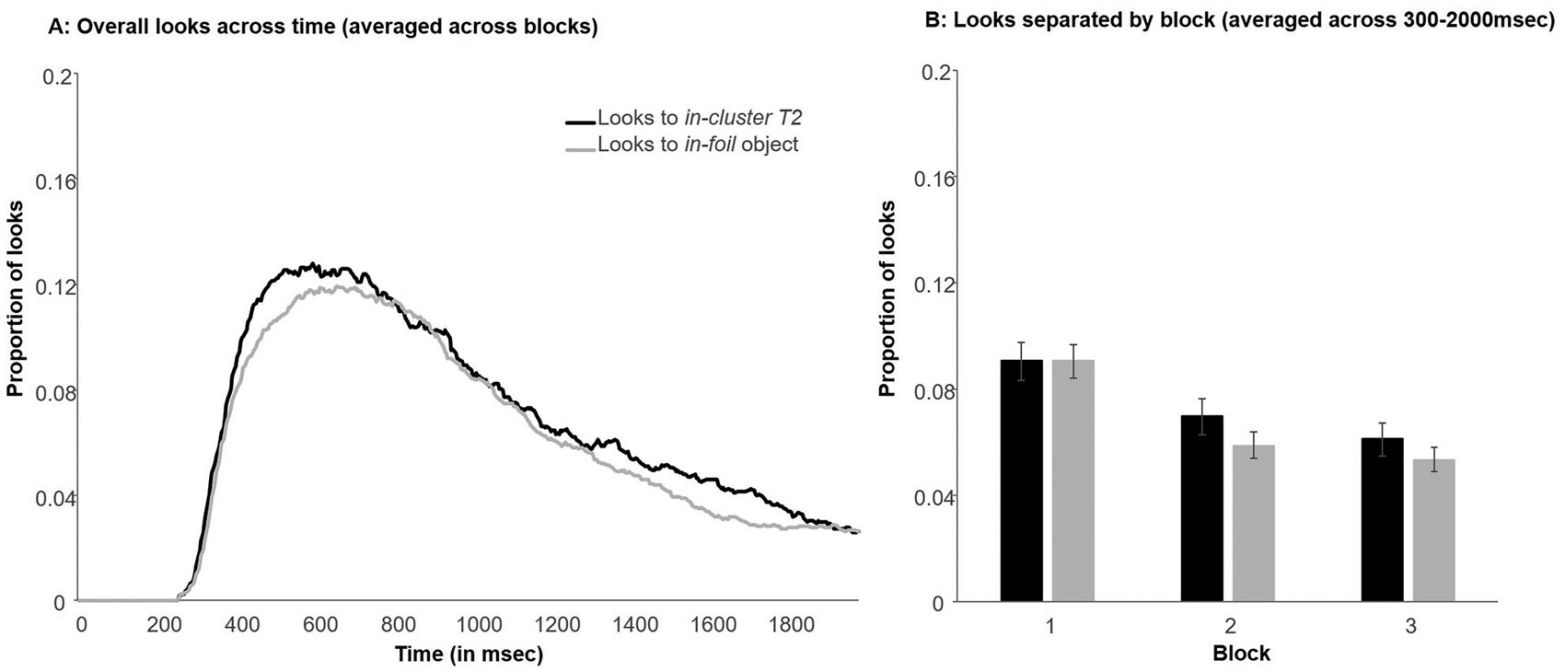
Looks during in‐cluster T2 trials of training in Experiment 1 across blocks and time (Panel A) and separated by block averaged across window of interest (300–2000 ms; Panel B). Error bars indicate the standard error of the mean. Looks to the target are not depicted to facilitate seeing differences between looks to the in‐cluster T2 and in‐foil object.

Next, we examined the eye movements during training. For this analysis, only correct trials were included. Again, participants’ behavior during *in‐cluster T2* training trials was investigated, testing whether they were more likely to look at an *in‐cluster T2* than a baseline foil (the average of the two baseline foils) even when they had selected the correct object (c.f., Roembke & McMurray, 2016). As is evident in Fig. 6A, participants looked slightly more to the *in‐cluster T2* than the *in‐foil* object during training. This effect appears to be more driven by differences in looks in Blocks 2 and 3 (Fig. 6B).

To examine this statistically, the looks for both the *in‐cluster T2* and the averaged baseline foils were calculated between 300 and 2000 ms for each participant (the area under the curve; Fig. 6B); looks that were initiated right before this window were excluded. This was analyzed in a linear mixed model with a fixed effect of block (1‐3; centered), object type (contrast coded; *in‐cluster T2* = 0.5, *in‐foil* = −0.5), and their interaction. The dependent measure was the proportion of looks to the *in‐cluster T2* and *in‐foil* between 300 and 2000 ms. The model that fit the data best only included a random intercept of subject (all other models had a singular fit).^4^

Using this model, the main effect of block was significant (*B* = −0.02, *SE* < 0.01, *t*(1848) = −8.62, *p* < .001). This indicates that participants made fewer eye movements to both T2 and foil objects as the experiment progressed. Moreover, the main effect of object type was significant, suggesting higher looks to the *in‐cluster T2* than the *in‐foil* competitor (*B* = 0.01, *SE* < 0.01, *t*(1847) = 2.06, *p* = .039). The interaction of block and object type was not significant (*B* < 0.01, *SE* < 0.01, *t*(1847) = 0.63, *p* = .526). The main effect of object type indicates that participants maintained small associations with the incorrect competitor throughout training (though the numerical difference in looks between the *in‐cluster T2* and baseline foils was small; see Fig. 6). This was surprising; therefore, to investigate the remaining associations further, we split training data by block and repeated the analyses with a model without the fixed effect of block and a random intercept for subject. After adjusting the *p*‐value with the Bonferroni correction (new α = 0.017), the effect of object type was not significant in any of the three blocks but was closest to significance in Block 2 (Block 1: *p* = .725; Block 2: *p* = .040; Block 3: *p* = .115).

#### 2.2.2 Testing

**Fig. 7:**
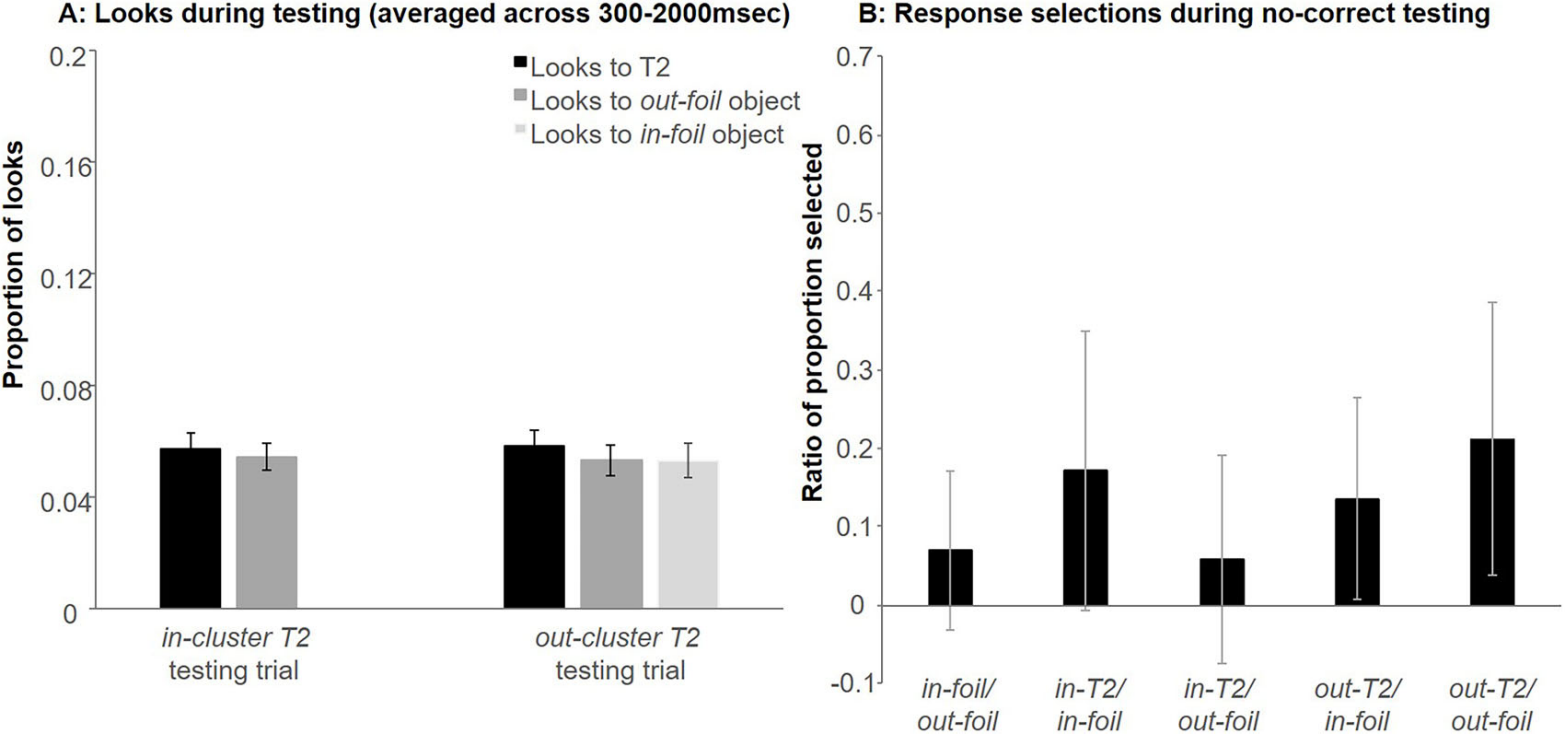
Looks during in‐cluster and out‐cluster T2 testing trials in Experiment 1 averaged across window of interest (300–2000 ms; Panel A) and responses during no‐correct testing (Panel B). Please note that in Panel B, we refer to the in‐cluster T2 and the out‐cluster T2 as in‐T2 and out‐T2 due to space restrictions. Error bars indicate the standard error of the mean.

Accuracy at test was high (*M* = 99%), so accuracy was not subjected to statistical analysis. The proportion of looks to the T2 and baseline foils was calculated between 300 and 2000 ms on correct trials only separately for *in‐cluster* and *out‐cluster T2* testing trials (Fig. 7A). We ran separate models for the *in‐cluster* and *out‐cluster T2* trials as these trials had different sets of relevant foils.

For *in‐cluster T2* testing trials, looks to the *in‐cluster T2* competitor were compared to the present baseline out‐foils. The statistical model always included a fixed effect of object type (contrast coded; *in‐cluster T2* = 0.5, *out‐foil* = –0.5). As before, the random effect structure was determined by comparing increasingly more complex nested models. Random effects analyses suggested that the model with only a random intercept of subject best fit the data.^5^ Participants did not look significantly more at the *in‐cluster T2* than the *out‐foils* (*B* < 0.01, *SE* < 0.01, *t*(597) = 0.583, *p* = .56).

For *out‐cluster T2* testing trials, there was also a fixed effect of object type; however, it had three levels (*out‐cluster T2*, *in‐foil*, *out‐foil*). Thus, object type was effect‐coded with each of the baseline foils being compared to the *out‐cluster T2* competitor. The model with only a random intercept of subject again best fit the data (more complex models resulted in a singular fit).^6^ However, neither comparison of object type reached significance (*out‐cluster T2* vs. *in‐foil*: *B* < −0.01, *SE* < 0.01, *t*(912.09) = −0.59, *p* = .555; *out‐cluster T2* vs. *out‐foil*: *B* < −0.01, *SE* < 0.01, *t*(912.09) = −0.50, *p* = .615).

#### 2.2.3 No‐correct testing

Finally, we examined selections during no‐correct testing (Fig. 7B). Two participants were excluded from the analysis as they selected the same response location more than eight times in a row, suggesting that they did not comply with task instructions. After converting proportion selections into log odds ratios, ratios were compared to zero (indicating no difference in frequency of selection) using a one‐sample *t*‐test. We compared the following contrasts: *in‐foil* versus *out‐foil*, *in‐cluster T2* versus *in‐foil*, *in‐cluster T2* versus *out‐foil*, *out‐cluster T2* versus *in‐foil*, *out‐cluster T2* versus *out‐foil*. None of the tested contrasts reached significance (all *M* < 0.25; all *p* > .100), indicating that participants did not “prefer” one response choice over another in the absence of the target.

### 2.3 Discussion

Participants’ selections during Block 1 of training indicated that they were sensitive to the pretraining co‐occurrence of words and T2s: They were significantly more likely to select the *in‐cluster T2* than a randomly selected *in‐foil*. This finding suggests that participants paid attention during pretraining and that pretraining affected their subsequent behavior during training. However, these learned additional associations were weak—if people had been matching the probabilities they observed during pretraining, they should have selected it 40% of the time, yet they did so at a rate of only 12% (averaged across Block 1). This suggests that these associations were either warped during pretraining (to favor the target over the T2), or that they were ignored or suppressed very rapidly at test (a point we return to in the General Discussion).

In contrast to this, eye‐tracking results suggest that participants maintained very small associations with the incorrect *in‐cluster T2* during training, and there was little evidence that this changed over the 336 training trials: there was no block × object interaction, and at the level of individual blocks, looks to the *in‐cluster T2* were most pronounced during Block 2 (though statistical evidence for this was not strong).

A pruning account would have predicted that incorrect associations were strongest during Block 1 (right after pretraining) and then became weaker as the correct associations were acquired. This was not observed. However, the relatively small effect in the eye movements is itself informative: negative associative learning may have occurred extremely rapidly. Given this, it may be that we simply cannot detect variation in the maintenance of the associations with the foil because such associations were at floor.

No increased looks to either of the T2s were observed during testing. That is, at test, both the *in‐* and *out‐cluster T2s* were not fixated more than baseline items. Thus, while these associations appear to have been present at Block 1 of training (as seen in the mouse click responses), and across training (vis à vis the eye movements), by the time of test, both were fully pruned. Consistent with this, the no‐correct testing session did not reveal any selection preferences of participants in the absence of the target object, again indicating that no weak associations between words and T2s were maintained after training.

Importantly, in contrast to what had been observed in pigeons (Roembke et al., 2016), this was even true for the *out‐cluster T2s* that had not co‐occurred with the words during training. This means that pruning can occur both on the basis of supervised feedback (*in‐cluster T2*) as well as in its absence (*out‐cluster T2*). What mechanisms could drive the latter? One possibility is the lack of co‐occurrence statistics as a form of cross‐situational learning—here, the fact that the target word never co‐occurred with the *out‐cluster T2* could be used as evidence that they should not be associated.

Overall, these data indicate that at least partial pruning was a relatively quick process, even for competitors that did not occur with the words during training (i.e., the *out‐cluster T2*). Thus, participants’ high learning rate may have facilitated the pruning of incorrect associations. Alternatively, the reduction of incorrect associations may have allowed participants to acquire word‐object‐mappings more quickly. We cannot draw any conclusions on the directionality of the effects, though, it is possible that the two are related.

If pruning is indeed such a quick elimination process, it would be hard to detect in an eye‐tracking paradigm that relies on the averaging of a number of trials (trials over which learning would have been actively weakening the effect). However, the pruning of incorrect associations may be slower if spurious associations are more pronounced; this should also facilitate observing the pruning process within the eye‐tracking paradigm.

Thus, Experiment 2 started by building stronger incorrect associations: In pretraining, words were now paired with their T2 on the majority of trials (80%) and with the target object on 20% of trials. It was predicted that participants would take longer to unlearn the incorrect associations to the T2, resulting in increased looks to the *in‐cluster T2* during the beginning of training. However, given how quickly participants were found to learn in Experiment 1, it was still predicted that pruning would be complete by testing.

## 3 Experiment 2

### 3.1 Method

#### 3.1.1 Participants

Forty‐two monolingual, native speakers of English with normal or corrected‐to‐normal vision participated in this experiment (19 females, 23 males, 0 nonbinary/preferred not to answer). All were adult students at the University of Iowa and received course credit as compensation for participation. No age information was collected for participants.

#### 3.1.2 Stimuli and design

The same design and materials were used as in Experiment 1 with one exception: During pretraining, each word was paired with their assigned target on a minority (20%) of trials (2/10 trials/word). At the same time, each word was paired with their T2 on 80% of all trials (8/10 trials/word). This was done to build a high amount of spurious (incorrect) associations with the T2, thus increasing the need to prune incorrect associations.

#### 3.1.3 Procedure, eye‐tracking recording, and analysis

The same procedure as in Experiment 1 was utilized. The same instructions were used as in Experiment 1. Eye movements were recorded using the same apparatus and analysis techniques as in Experiment 1.

### 3.2 Results

#### 3.2.1 Training

Three participants were excluded, two because of construction noise during the experiment and a third quit the experiment after two blocks of training.

Performance on the training trials during training is presented in Fig. 5: Participants were much slower to learn the word‐object‐mappings when pretraining favored the T2 instead of the eventual target object (e.g., Block 1, *M* = 53%). We analyzed participants’ responses on *in‐cluster T2* training trials of Block 1 to ask if the *in‐cluster T2* was selected more often than a baseline foil: The target was selected on 45% of trials, the *in‐cluster T2* on 34%, and the *in‐foil* on 12% (average of the two foils) of the trials. An odds ratio analysis showed that the difference between the rate of selecting the *in‐cluster T2* and the *in‐foil* was significant (*t*(38) = 10.44, *p* < .001). Thus, there was only a slight increase in how often the *in‐foil* was selected in comparison to Experiment 1 (8%), but participants’ selection between the *in‐cluster T2* and the target object was more evenly split. This is further evidence that participants were highly sensitive to the co‐occurrence statistics during pretraining. In Block 2, the *in‐cluster T2* was selected on 10% of trials (*in‐foil* = 6%). In Block 3, the *in‐cluster T2* was selected on 5% of trials (*in‐foil* = 2%). The difference in selection rate was marginally significant in Block 2 (*p* = .056) but significant in Block 3, suggesting the maintenance of small associations between target word and *in‐cluster T2* during training (*t*(38) = 3.51, *p* = .001).

**Fig. 8:**
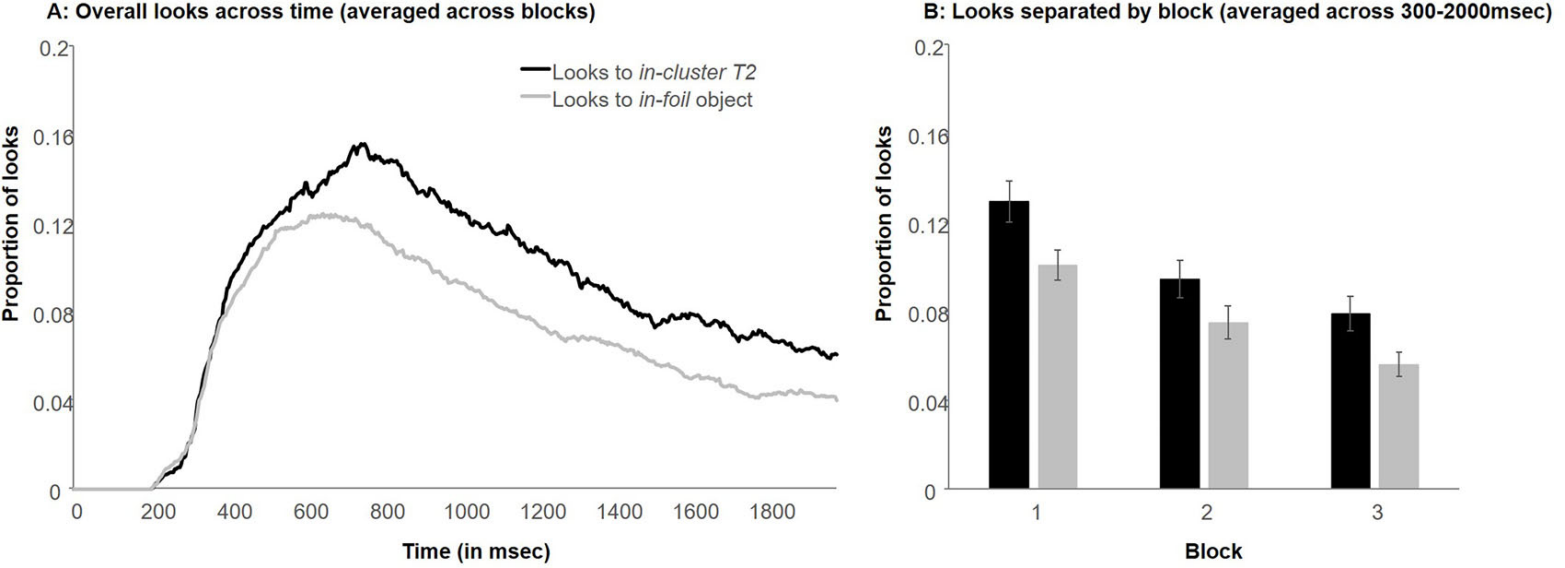
Looks during in‐cluster T2 trials of training in Experiment 2 across blocks and time (Panel A) and separated by block averaged across window of interest (300–2000 ms; Panel B). Error bars indicate the standard error of the mean. Looks to the target are not depicted to facilitate seeing differences between looks to the in‐cluster T2 and in‐foil object.

Next, eye movements during training were analyzed. Eye‐tracking data from training can be seen in Fig. 8. This depiction suggests that participants may have retained (relatively strong) incorrect associations throughout training, even as they learned the correct word‐object‐mappings. Interestingly, looks to the T2 appear to be above the *in‐foil* even at the end of the trial (Fig. 8A); this suggests that the T2 may never get fully suppressed during real‐time processing. Similarly, looks to the *in‐cluster T2* remain high across blocks (Fig. 8B).

To investigate this statistically, the proportion of looks to each object type was calculated on correct *in‐cluster T2* trials between 300 and 2000 ms. The fixed effects were object type (contrast coded; *in‐cluster T2* = 0.5, *in‐foil* = −0.5) and block (centered). The model that best fit the data included a random intercept for subject with no random slopes^7^; none of the more complex models converged. Participants’ overall number of looks decreased as the experiment progressed (*B* = −0.03, *SE* = 0.03, *t*(1633) = −8.88, *p* < .001), consistent with Experiment 1 and standard eye‐tracking findings. There was also a main effect of object type, indicating that participants were significantly more likely to look at the *in‐cluster T2* than the *in‐foil*, independently of the block number (*B* = 0.03, *SE* < 0.01, *t*(1626) = 6.35, *p* < .001). In addition, the interaction between block and object type was not significant (*B* < −0.01, *SE* = 0.01, *t*(1626) = −1.53, *p* = .127). This relatively stable looking to the *in‐cluster T2* suggests that the increased co‐occurrence of the T2 and the word during pretraining were strong enough to act as a “protector” against quick pruning.

#### 3.2.2 Testing

**Fig. 9:**
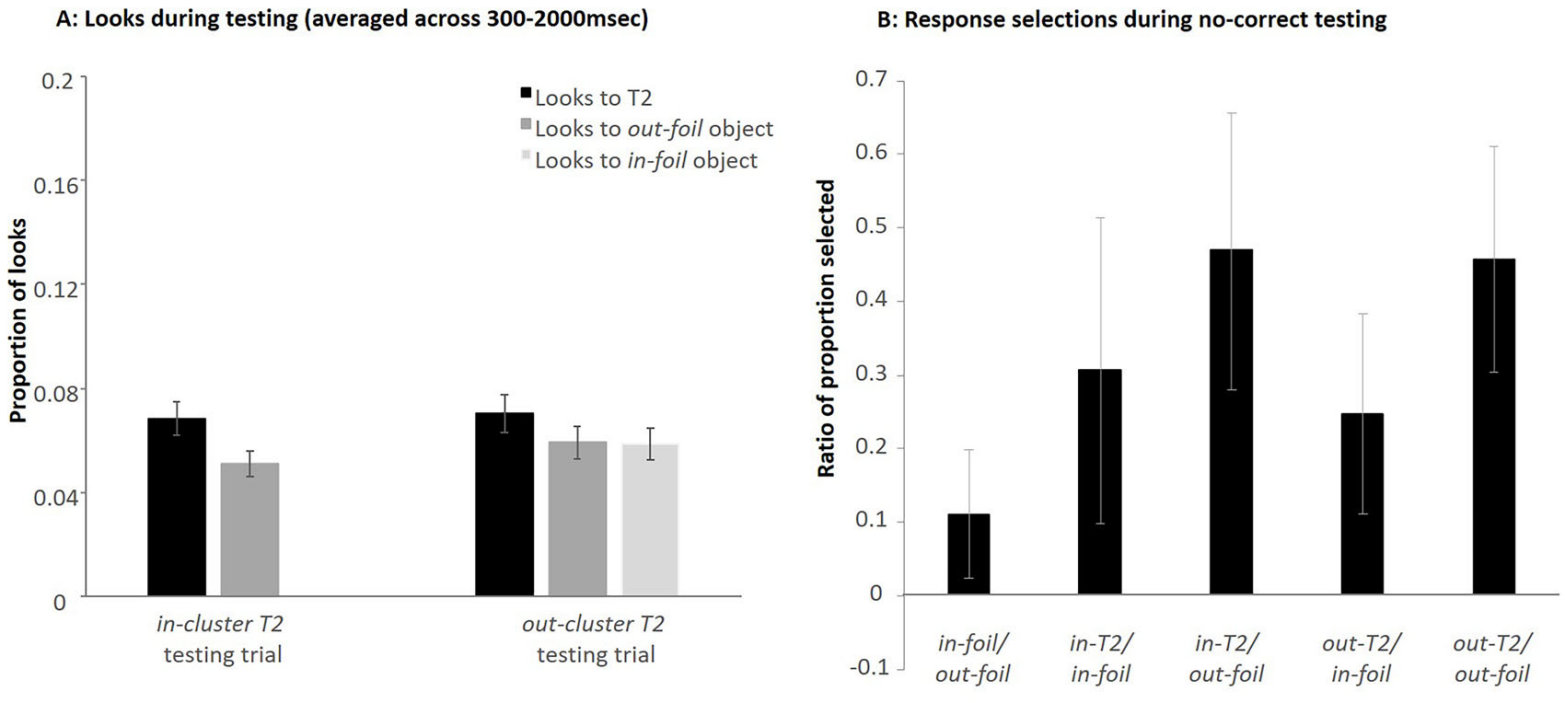
Looks during in‐cluster and out‐cluster T2 testing trials in Experiment 2 averaged across window of interest (300–2000 ms; Panel A) and responses during no‐correct testing (Panel B). Please note that in Panel B, we refer to the in‐cluster T2 and the out‐cluster T2 as in‐T2 and out‐T2 due to space restrictions. Error bars indicate the standard error of the mean.

Participants performed close to ceiling at testing (*M* = 96%). As can be seen in Fig. 9A, looks to T2s remained higher than baseline foils during testing. As before, *in‐cluster T2* trials were analyzed separately from *out‐cluster* ones.

For *in‐cluster T2* testing trials, the model that best captured the data again included only a random intercept of subject (the simplest model tested). This model showed a significant effect of object type (contrasted coded as before), indicating that participants were more likely to look to the *in‐cluster T2* than the baseline *out‐foils* (*B* = 0.02, *SE* = 0.01, *t*(580.2) = 3.18, *p* = .002).

For *out‐cluster T2* testing trials, again two effect‐coded contrasts were formed as in Experiment 1 to compare looks to the *out‐cluster T2* with *in‐foils* and *out‐foils*, respectively, as object type was effect‐coded with the T2 as the object to which baseline looks were compared. As before, the model with only a random intercept of subject fit the data best^8^ (fit was singular for more complex models). Neither the comparison of looks to the *out‐cluster T2* and the *in‐foil* nor the comparison of looks to the *out‐cluster T2* and the *out‐foil* were significant (*out‐cluster T2* and *in‐foil*: *B* < −0.01, *SE* < 0.01, *t*(886.21) = −1.83, *p* = .067; *out‐cluster T2* and *out‐foil*: *B* < −0.01, *SE* < 0.01, *t*(886.21) = −0.55, *p* = .581).

#### 3.2.3 No‐correct testing

For no‐correct testing, one additional participant's data could not be included, as they did not complete this section due to time limitations. For each trial type, the proportion of competitor selection were calculated and transformed into log‐odds ratios. Ratios were compared against 0 using a one‐sample *t*‐test; values above zero indicate that the numerator of the ratio was selected more often than the denominator (Fig. 9B). Participants were equally likely to select the *in‐foil* and the *out‐foil* during control trials (*t*(37) = 1.26, *p* = .108). The T2 was more frequently chosen than any other competitor type. This was true for both *in‐cluster T2s* as well as *out‐cluster T2s* (*in‐cluster T2*/*out‐foil*: *t*(37) = 2.50, *p* = .008; *out‐cluster T2*/*in‐foil*: *t*(37) = 1.86, *p* = .0345; *out‐cluster T2*/*out‐foil*: *t*(37) = 2.86, *p* = .003). The one exception was the *in‐cluster T2*/*in‐foil* comparison which was marginally significant: *t*(37) = 1.50, *p* = .071. These findings are consistent with the eye‐tracking results, suggesting that participants did not prune incorrect associations to *in‐cluster T2s* during training. This lack of pruning was even true for the *out‐cluster T2*, which never co‐occurred with the target word during training (and despite the nonsignificant eye‐tracking findings as part of the *out‐cluster T2* testing trials analysis).

### 3.3 Discussion

The increased co‐occurrence of the T2 with its target word during pretraining led to a much more robust association between them. This was seen both in the responses to the foil during training and in the fixations. Intriguingly, these associations were never fully pruned: Participants continued to fixate the T2s during training, even as they acquired the correct word‐object‐mappings.

Moreover, at test, participants more strongly activated the *in‐cluster T2* in testing trials, and selected both the *in‐cluster* and the *out‐cluster T2* more often than other competitor types during no‐correct testing. This stands in contrast to the findings of Experiment 1, where pruning was completed within training. Again, the only difference between the two studies was the co‐occurrence statistics during pretraining: During Experiment 1's pretraining, the word was presented with its target object on 60% of all trials and with the T2 on the remaining 40%. However, in Experiment 2, pretraining involved the word's presentation with the T2 on 80% of all trials, thus leaving only 20% of co‐occurrence with the target object.

The results of Experiment 2 suggest two conclusions: First, pruning is not always quick and efficient, but can be prolonged or even avoided. That is, even as listeners achieved near‐ceiling accuracy, they continued to fixate the *in‐cluster T2* at above chance levels. Under what circumstances then, are associations pruned, and under which ones are they more “protected” (i.e., under which circumstances is pruning more difficult)?

It is possible that participants’ explicit (conscious) knowledge of the word‐object‐mappings played a role (which likely is impacted by the strength of the trained associations). After Experiment 2's pretraining, participants may have been aware of which object each word (presumably) mapped onto most strongly (i.e., the T2 which was more probable than the eventual target). During training and testing, the incorrect associations between the word and the T2 may have been maintained through participants’ explicit memory of pretraining (potentially even as associative pruning took place). In contrast, in Experiment 1, participants may have not been consciously aware of the word more often appearing with its target during pretraining due to the relatively even split between the two objects. At this point, this explanation is speculative. Nevertheless, it is consistent with findings from cross‐situational word learning that how words are learned is impacted by whether people develop conscious awareness of mappings or not (Wang, 2020). Importantly, if pruning is impacted by participants’ higher‐level processing, it would not be possible to observe such interactions in less‐developed species such as pigeons.

Second, Experiment 2 further supports the notion that pruning operates similarly for the *in‐cluster* as well as the *out‐cluster T2*: The results differed across experiments, but were similar within an experiment across the two types of competitors: either associations with both are pruned (Experiment 1) or not (Experiment 2). This is surprising, considering the findings in pigeons by Roembke et al. (2016), where pruning was dependent on the negative feedback received after incorrect selections. That is, pruning consistently only occurred in the birds when T2s were experienced within the same cluster during learning.

In contrast, it seems that in humans, negative associative learning may be more robustly related to unsupervised statistical learning processes: If, as in pigeons, supervised learning was driving pruning, we should have seen decreasing consideration of the *in‐cluster T2*, while the *out‐cluster T2* should have remained a viable target even after training (also see Fig. 4 for clarification). Instead, it appears that the strength of the original spurious association determined whether negative associative learning took place or not. Moreover, note that eye‐movement evidence for activation of the *out‐cluster T2* at test was weaker than for the *in‐cluster T2*. Again, this supports a potentially stronger role for unsupervised learning (which pruned the *out‐cluster T2*) than supervised learning (which would have pruned the *in‐cluster*, given the more robust evidence for its consideration).

Overall, Experiment 2 indicates that pruning may be a more complex process than previously assumed: If a word is presented with an incorrect referent repeatedly, people struggle with unlearning this alternative “hypothesis,” even as they correctly select the target object.

## 4 General discussion

### 4.1 Overview of results

The goal of this study was to determine whether incorrect associations between words and objects are pruned as the result of supervised and/or unsupervised learning. For this purpose, participants were first trained on two referents for each word, and subsequently, tested whether the incorrect meaning was maintained even after receiving consistent feedback that the word only had one correct meaning after all.

Critically, one of these classes of referents (the *in‐cluster T2*) was repeatedly experienced during training (as a foil). This would have afforded the opportunity for supervised learning to participate in pruning, even as the co‐occurrence statistics could have helped maintain its association with the target word. The other class of referents (the *out‐cluster T2*) was never experienced on the same trial as the word; consequently, it never could have undergone supervised learning, but it had a co‐occurrence of 0 with the relevant target word, allowing unsupervised mechanisms to prune these associations.

Experiment 1 found that some pruning of incorrect associations proceeded relatively quickly. By the first block of training, responding to the *in‐cluster T2s* was very low (though significantly more than the foils), and while the fixations suggested some association, the overall difference was quite small. There are several possible mechanisms for these early effects. First, it is possible that this is not the result of pruning: perhaps participants never really acquired the association. That is, even though the target object was only slightly more likely to co‐occur with the target word than the T2, participants may have only acquired the stronger of the two associations. Alternatively, participants may have had the incorrect associations but just partially ignored them to the extent that only little looks were exerted to the T2s (as some kind of a top‐down strategy); or they may have rapidly lost the incorrect associations during training (i.e., they were pruned).

Longer term, however, we also observed a slower pruning process. Throughout the training period, the *in‐cluster T2* appeared to be preserved, and it was not until after training that participants no longer activated the incorrect referent that they had been previously trained on (as indicated by the eye‐tracking and no‐correct testing data). The loss of the association (going into the testing trials) cannot derive from a failure to fully learn the association (as there was small evidence for this earlier in training). So, does this represent true associative pruning or a top‐down effect? The low selection of the *in‐cluster T2* in combination with the small eye‐tracking effect during training (slightly more looks to the *in‐cluster T2* than baseline foils) is consistent with the use of a top‐down strategy; at the same time, the fact that there were no looks to the T2s during testing and no above‐baseline selection during no‐correct testing suggests complete pruning.

Experiment 2 also showed some evidence for rapid pruning by Block 1 (even as it was slower than in Experiment 1, since the participants started with stronger associations). However, it showed that the secondary meaning was not fully suppressed even at the end of the experiment (as indicated by more looks to the *in‐cluster T2* during testing and above‐baseline selection of both T2s during no‐correct testing, even as participants learned word‐object‐mappings within approximately two blocks of training).

Interestingly, pruning was more effective when unsupervised statistics did not support an association between a word and referent: When the T2 was assigned to the opposite cluster than the word (the *out‐cluster T2*), participants never activated words at testing (in both experiments), despite the fact that this object never received any negative reinforcement during training. In contrast, when the T2 was part of the cluster, it was robustly maintained despite the opportunities for negative reinforcement during training. This suggests that feedback was not sufficient for pruning incorrect associations. Rather, this is more likely to be driven by unsupervised processes based on co‐occurrence. Here, the *out‐cluster T2* had much more robust statistical evidence against it (since its co‐occurrence with the target word was effectively zero), while the *in‐cluster T2* frequently co‐occurred with the target during training (on 3/7 of trials ≈ 43%), in order to create opportunities for negative reinforcement during training.

One caveat to this interpretation is that participants’ accuracy during learning was often very high. This was required for the logic of our eye‐tracking analysis which only analyzed correct trials. However, as a result of this, participants did not receive much negative reinforcement, since they were typically correct. In fact, they most often received confirmatory feedback after selecting the target object. This stands in contrast to the pigeons from Roembke et al. (2016) study, where learning was much slower and negative feedback was frequent. However, we point out that most supervised learning rules (e.g., Rescorla & Wagner, 1972) adjust associations between the stimulus and all available responses, even if not selected, the foil would receive some negative learning. Nonetheless, it is possible that we would observe more robust reinforcement‐driven pruning with a design that had a higher error rate.

Finally, an alternative interpretation in this paradigm was that participants came to see the T2 as not an incorrect choice, but rather as a secondary meaning of the target word. This is particularly likely for the *out‐cluster T2s*, as they never underwent any negative reinforcement which could rule out the possibility of a secondary meaning. However, were this the case, one might predict that at test when the *out‐cluster T2* is suddenly available (particularly on the no‐correct trials), it might “spring to life,” receiving a lot more fixations and overt responses. However, this was not observed, and if anything the *out‐cluster T2* showed less evidence for being retained than the *in‐cluster*.

### 4.2 Interactions of supervised and unsupervised pruning during word learning

It is well‐established that people use co‐occurrence information to learn novel words (e.g., Dautriche & Chemla, 2014; Fitneva & Christiansen, 2017; Poepsel & Weiss, 2016; Roembke & McMurray, 2016; Scott & Fisher, 2012; Smith & Yu, 2008; Suanda, Mugwanya, & Namy, 2014; Yu & Smith, 2007), and that learners can form multiple associations for the same target word (e.g., Roembke & McMurray, 2016; Yurovsky et al., 2014). The present study further supports the notion that participants maintain multiple hypotheses for the same word (Roembke & McMurray, 2016), even as they have no issue retrieving the correct target (as indicated by the overall high performance). In this regard, even in Experiment 1, the eye‐movement data suggests the T2 maintained a small association throughout training, and Experiment 2 found particularly robust evidence that the T2 continued to be associated. Further, this experiment adds that these associations may also undergo pruning on the basis of statistical learning. This kind of flexible maintenance of the lexical network is essential for a more robust vocabulary, and is not strongly consistent with at least existing hypothesis‐testing accounts of vocabulary acquisition (Medina et al., 2011; Trueswell et al., 2013). Moreover, as we discuss in 4.3, this kind of flexible learning may be useful and functional for a number of situations.

One open question remains whether a certain level of associative strength protects from pruning (as potentially observed in Experiment 2): if one has learned that a specific furniture piece is a *sofa*, it may be functional to retain that word‐object mapping even if one later learns that it can also be referred to as a *couch*. Similarly, for learners of a second language, the strength of the known label (in the first language) is likely well‐established, so that it is not pruned even if a second one is acquired. In addition, it is unclear whether pruning of incorrect associations between words and T2s was complete, or if small amounts remained. Here, we see mixed evidence: while Experiment 2 shows clear evidence for retention of the *in‐cluster T2* over the course of training and through testing, Experiment 1 showed much more rapid pruning. However, even in Experiment 1, it may be that these associations were not completely lost and may remain “functional” (we will come back to this point in the subsequent section).

One surprising result is the interaction of unsupervised and supervised mechanisms during word learning. All supervised experiments also include unsupervised statistics (word/object co‐occurrences) that could impact learning. Even as human participants received feedback that the *in‐cluster T2* was not the target, they could have been tracking its co‐occurrence with the target word, helping to maintain the association. This is consistent with the finding that learners also encode associations among the novel objects during cross‐situational word learning (Zettersten, Wojcik, Benitez, & Saffran, 2018). The inclusion of unsupervised statistics is in some ways an artifact of not all objects appearing on any trial—however, this is almost certainly a property of any real‐world learning interaction.

The fact that two items did not appear together also carries important information, even if no feedback was given that the two did not map onto each other. That is, particularly in Experiment 2, the *out‐foil* received very little consideration at test. This implies that unsupervised statistics—at least in the context of human word learning—may outweigh supervised ones when it comes to negative associative learning. At the same time, it also raises the possibility that whether participants are explicitly aware could impact how associations are pruned. Future research should directly test these interactions between different types of statistical regularities (Zettersten et al., 2018) and consciousness, and examine whether this conclusion generalizes to other areas of learning and/or populations (e.g., children).

### 4.3 Implications for vocabulary acquisition and avenues for future research

At the broadest level, our study supports the notion that both supervised and unsupervised statistics interact during adult word learning (as discussed in the previous section). Beyond that, one question that our study raises is to what extent these spurious associations are really “incorrect” but rather are a functional component of the lexicon. While we have treated these T2 associations as simply “incorrect,” in the real world, there may be a number of more nuanced situations where they arise.

First, many objects have multiple words (e.g., *couch/sofa*, *cup/glass*). However, over the course of learning, one could be pruned (via the statistical mechanisms learned here). For example, when a child is repeatedly exposed to *sofa*, they may have to partially relearn *couch* when they are in a context where that word is the more common way to refer to it. It is possible that the presence of a latent association helps when relearning the mapping between a word and its T2 (see Yurovsky et al., 2014, for evidence in this direction). This could be particularly important as learners flexibly sort out synonyms and near synonyms.

Second, the small associations between targets and T2s could contribute to word‐word (semantic) associations in the lexicon (e.g., *nurse* and *doctor* are semantically associated) and influence word recognition in‐the‐moment. In fact, it is well‐established using a similar eye‐tracking paradigm as here (i.e., the VWP) that people also activate semantic competitors during spoken word recognition (Huettig & Altmann, 2005; Jeppsen, Baxelbaum, Tomblin, Klein, & McMurray, 2024; Yee & Sedivy, 2006). That is, even if these associations are wrong, their presence may contribute to a broader ability to recognize the discourse or general semantic context, priming words/referents that are likely to arise.

It has been argued that young children—in contrast to adults—are more associative learners (Ramscar, Dye, & Klein, 2013). This may be because the medial temporal lobe is developing relatively late in childhood (Townsend, Richmond, Vogel‐Farley, & Thomas, 2010), so that children may rely more on nondeclarative memory systems during early vocabulary acquisition. This is supported by work with hippocampal amnesiacs that suggests that cross‐situational learning is possible (though impaired) even in the absence of such systems (Warren, Roembke, Covington, McMurray, & Duff, 2020). As such, it is not unreasonable to speculate that children's slower learning and pruning may be more in line with what was observed in pigeons (Roembke et al., 2016). If this were the case, pruning in children may be more consistent with a supervised account than what we observed here.

Moreover, in recent debates of word learning, one argument that has been made is that children wait for “diamonds”—situations in which it is very clear which object was named, thus facilitating the formation of a word‐object‐mapping and overcoming referential ambiguity (e.g., in the context of cross‐situational word learning: Medina et al., 2011; Trueswell et al., 2013). A similar argument could be made in the context of unlearning of incorrect associations, proposing that specific situations where an incorrect association is explicitly rejected are needed for unlearning. If such an account of word learning is adopted, unlearning of incorrect associations is even more important because each association that is formed is stronger.

At the same time, when we consider the role of pruning in the broader lexical network (considering things like synonyms, priming, etc.), a simple one‐shot all or nothing form of pruning (or learning) seems less likely to account for the flexibility and complexity of the adult lexical network than a more gradual statistically sensitive form of learning. This highlights the importance of better understanding the level of incorrect associations that naturally form, and what experiences are necessary to prune misleading mappings.

### 4.4 Limitations

Several limitations of this work should be considered: First, it is possible that looks to the T2s at test (for both types of competitors in Experiment 1, and for the *out‐cluster* T2s in Experiment 2) were not statistically detectable due to the smaller number of trials used for analysis (in comparison to training trials). However, this is unlikely, given that numeric differences between looks to the different foil types were very small if they existed at all.

Second, we used eye‐tracking in the VWP to detect subtle differences in how different word meanings were activated during processing. However, this methodological approach may make it difficult to measure differences in activation strength over shorter sequences of training trials. This is the case, as the VWP relies on the averaging across many trials. As a result, it is possible that participants looked slightly more to the T2 (e.g., early in training in Experiment 1), even if it did not appear in their eye movements. In addition, it should be noted that eye‐tracking is a relatively conservative measure of activation strength, as only trials in which the correct object was selected are analyzed. Trials in which the T2 was selected—the strongest evidence that the secondary meaning was activated—are not considered (Roembke & McMurray, 2016). This is why participants were found to select T2s more than the baseline foils during Block 1 of training in Experiment 1, even as no differences in looks were detected.

Third, our study does not speak to the exact real‐life situations in which and age groups for which pruning may take place. To be clear, this was never our goal; rather, we were hoping to find proof‐of‐concept for pruning in human learners. In the context of this experiment, the associations with the *in‐cluster T2* are explicitly defined as incorrect (participants receive negative feedback when it is selected); for the *out‐cluster T2*—as described earlier in detail—its “incorrectness” is more implicit due to being consistently reinforced with another word. In addition, the objects that were used as referents were not visually similar in a way that suggested semantic relatedness; that is, any semantic closeness was established based on the co‐occurrence of words and objects.

As such, our study is most closely related to a situation in which an incorrect association was established first and then revised. This could occur both during children's but also adults’ word learning. For example, a toddler may first learn to associate the word *duck* with ducks and ponds, but may later revise that to just refer to the bird or they may erroneously conclude that *fork* refers to the vase of flowers that is present at every dinner. But these kinds of spurious associations are not limited to childhood; adults may encounter new words in educational contexts or due to cultural changes that may need to be unlearned. It is likely that some kind of pruning also occurs in other learning contexts, such as second language acquisition. Here, however, “old” (first language) associations would not be incorrect but rather remain valid, even as new (second language) associations are added. This could be particularly complex in language‐switching situations where a new word could be in either language. Again, this speaks to the need for avoiding a strong and complete pruning. Future research needs to qualify how pruning, if at all, may occur in these contexts where two (or more) associations may receive positive reinforcement inconsistently.

### 4.5 Conclusion

This study offers a first step in investigating whether the unlearning of incorrect associations is a critical part of word learning. We conclude that incorrect meaning associations between words and objects are pruned, though, the extent to which this happens depends on the strength of the incorrect associations. Further, our data suggest that supervised and unsupervised mechanisms interact during (un)learning, with unsupervised information (co‐occurrence statistics) outweighing supervised one (feedback) under some circumstances.

## Notes

1 We understand that such semantic associations may not actually be “incorrect” per se but rather represent an integral part of semantic memory. We return to this point in the General Discussion.

2 Each experiment additionally included a word recognition phase after no‐correct testing. However, these data were exploratory and will not be discussed in this manuscript.

3 The participant was only aware of these blocks in the sense that they were offered a small break between them. Nonetheless, block was a unit of analysis and design. We used it a proxy for time in this design, with participants being expected to learn as time proceeds. Moreover, for the VWP, it is necessary for trials to be averaged across a certain number of trials; otherwise, it is not possible to calculate the proportion of looks. The exact number of trials per block for the different experiment sections was decided based on both previous experiments (defining a lower boundary) and the needs of the clustering design. This was decided a priori and incorporated into the trial randomization.

4 In lmer() notation, this model is given by Looks ∼ object‐type * block + (1 | subject).

5 In lmer() notation, this model is given by: Looks ∼ object‐type + (1 | subject).

6 In lmer() notation: Looks ∼ object‐type + (1 | subject).

7 In lmer() notation: Looks ∼ object‐type*block + (1 | subject).

8 In lmer() notation: Looks ∼ object‐type + (1 | subject).
